# Evolutionary genomics of host-use in bifurcating demes of RNA virus phi-6

**DOI:** 10.1186/1471-2148-12-153

**Published:** 2012-08-22

**Authors:** Paul E Turner, Robert C McBride, Siobain Duffy, Rebecca Montville, Li-San Wang, Yul W Yang, Sun Jin Lee, Junhyong Kim

**Affiliations:** 1 Department of Ecology and Evolutionary Biology, Yale University, New Haven, CT 06520, USA; 2 Department of Pathology, University of Pennsylvania, Philadelphia, PA 19104, USA; 3 Department of Biology, University of Pennsylvania, Philadelphia, PA 19104, USA; 4 Current address: Sapphire Energy, Inc., 3115 Merryfield Row, San Diego, CA 92121, USA; 5 Current address: Department of Ecology, Evolution and Natural Resources, Rutgers, The State University of New Jersey, New Brunswick, NJ 08901, USA; 6 Current address: Stanford School of Medicine, 300 Pasteur Drive, Stanford, CA 94305, USA

**Keywords:** Adaptation, Bacteria, Bacteriophage, Experimental evolution, Known phylogeny, *Pseudomonas*, Virus

## Abstract

**Background:**

Viruses are exceedingly diverse in their evolved strategies to manipulate hosts for viral replication. However, despite these differences, most virus populations will occasionally experience two commonly-encountered challenges: growth in variable host environments, and growth under fluctuating population sizes. We used the segmented RNA bacteriophage ϕ6 as a model for studying the evolutionary genomics of virus adaptation in the face of host switches and parametrically varying population sizes. To do so, we created a bifurcating deme structure that reflected lineage splitting in natural populations, allowing us to test whether phylogenetic algorithms could accurately resolve this ‘known phylogeny’. The resulting tree yielded 32 clones at the tips and internal nodes; these strains were fully sequenced and measured for phenotypic changes in selected traits (fitness on original and novel hosts).

**Results:**

We observed that RNA segment size was negatively correlated with the extent of molecular change in the imposed treatments; molecular substitutions tended to cluster on the Small and Medium RNA chromosomes of the virus, and not on the Large segment. Our study yielded a very large molecular and phenotypic dataset, fostering possible inferences on genotype-phenotype associations. Using further experimental evolution, we confirmed an inference on the unanticipated role of an allelic switch in a viral assembly protein, which governed viral performance across host environments.

**Conclusions:**

Our study demonstrated that varying complexities can be simultaneously incorporated into experimental evolution, to examine the combined effects of population size, and adaptation in novel environments. The imposed bifurcating structure revealed that some methods for phylogenetic reconstruction failed to resolve the true phylogeny, owing to a paucity of molecular substitutions separating the RNA viruses that evolved in our study.

## Background

Viruses are powerful and relevant models for understanding fundamental molecular biology, genetics and evolution [1-3], and elucidating infectious-disease evolution [4,5]. The typically short generation times, large population sizes, and high mutation rates of RNA viruses make such studies highly efficient from an evolutionary standpoint [6]. Furthermore, the small genome sizes and disease importance of RNA viruses make them particularly attractive for research in evolutionary genomics of virus-host interactions, such as molecular evolution of virus speciation events [7], and divergence in viral genetic architectures due to host specialization versus generalization [8,9]. Previous experimental evolution studies with viral models generally used either individual populations or homogeneous spatially structured experimental design. More complex demographics such as a phylogenetic tree structure have been studied but generally under a small set of selective conditions [10-12]. Here we examine how RNA viruses evolve with demographic structure imposed by a phylogenetic tree, under selective conditions of novel host environments and parametrically varying population sizes.

Viruses are exceedingly diverse in their evolved strategies to manipulate hosts for viral replication [13-15]. Despite this diversity, variable environments and fluctuating population sizes are two challenges often faced by virus populations. First, viruses can be passively transmitted between hosts (e.g., via aerosols, fluids and vectors) and cannot evaluate host ‘habitat quality’ prior to infection [16], creating the possibility that virus particles bind to less permissive target cells (e.g., hosts of low ‘quality’ for virus reproduction). Therefore, virus populations may unexpectedly encounter new environments, including intrahost changes brought on by immune function [17,18]. Second, virus population size naturally tends to fluctuate, sometimes by several orders of magnitude [19-21]; e.g., virus population size will necessarily vary due to changing availability of susceptible host individuals, occurrence of transmission bottlenecks when initiating a new infection, and immunity-related fluctuations in within-host viral load. These variable population sizes can lead to differing relative strengths of natural selection versus genetic drift acting in virus evolution [22].

### Experimental overview

**Figure 1:**
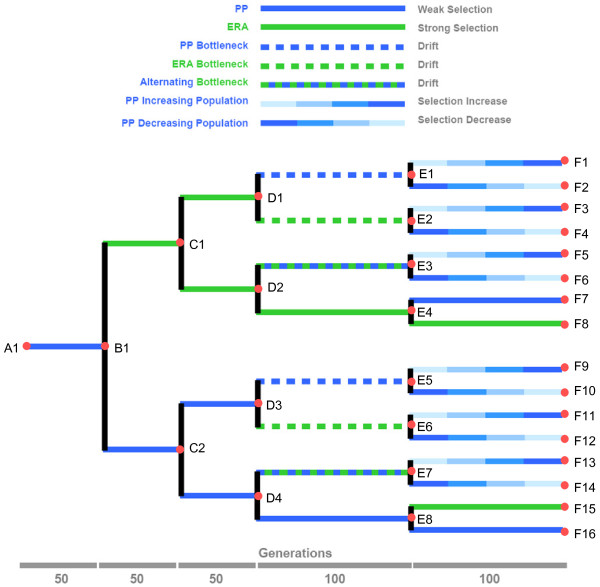
**Design for experimental evolution of phage ϕ6 populations propagated via bifurcating demes, under various host-use challenges and population sizes.** Labeled root, nodes and tips of the tree indicate isolated clones, subjected to genome sequencing

We examined the experimental evolution of RNA bacteriophage (phage) ϕ6 by tracking molecular and phenotypic changes in virus lineages experimentally evolved under environmental variation and fluctuating population sizes. Figure 1 shows our experimental design: a bifurcating deme structure that reflects lineage splitting that gives rise to demes with separated gene pools. Thus, we created a ‘known phylogeny’, allowing tests of the accuracy of phylogenetic algorithms [11,23]. Phage ϕ6 is typically grown in the laboratory on the plant pathogenic bacterium *Pseudomonas syringae* pathovar *phaseolicola*, but prior work [24] shows that the virus can adaptively improve on this host. However, selection pressures differ and greater adaptive change occurs when phage ϕ6 is cultured on the novel host *P. pseudoalcaligenes *[7,16,25,26], which is distantly related to *P. phaseolicola *[27,28]. Here we extended this work by contrasting phage ϕ6 evolution on the original and novel hosts and considering effects of host switching on virus evolution. Previous studies examined the effects of population size on mutational load and subsequent fitness recovery in phage ϕ6 [29,30]. Here we examined how constant versus variable population sizes impacted host-use adaptation in the virus. Overall, by combining whole-genome sequencing and measurements of host-use traits, we generated a very large dataset that fostered subsequent tests of inferred genotype-phenotype associations.

The known-phylogeny experiment, with additional independent confirmation using experimental evolution, demonstrated that host-use adaptation in phage ϕ6 involved an allelic switch in the viral assembly protein, a locus previously unknown to function in host-specific growth of the virus. More generally, our combined genomic/phenotypic approach was used to show where molecular substitutions tended to cluster in the genomes of phage ϕ6 populations subjected to the host-use challenges. Last, we showed that several popular methods were unable to accurately resolve the true experimental phylogeny, due to a paucity of molecular substitutions separating the sequenced clones.

## Results and discussion

### Molecular evolution

Phage ϕ6 has a ~13 kb segmented tri-partite dsRNA genome [31,32]. The three genomic segments are denoted Large (L; 6374 bp), Medium (M; 4063 bp), and Small (S; 2948 bp). The genome is organized such that polymerase functions are located on segment L, genes for host attachment proteins are on segment M, and genes for the nucleocapsid shell (P8), the major membrane protein (P9), the lytic enzyme (P5), and the membrane assembly nonstructural protein (P12) are on segment S [32]. Segment reassortment can occur when multiple ϕ6 viruses co-infect the same host cell and generate reassortant (hybrid) progeny, which contain a mixture of the segments found in the co-infecting parents [33,34]. However, recombination (breaking and joining of homologous RNA segments) is lacking or occurs at an extremely low rate in phage ϕ6 [33], allowing its possible effects to be ignored in our study.

We challenged phage ϕ6 lineages to evolve on their typical host *P. phaseolicola* (PP), and on the distantly-related novel host *P. pseudoalcaligenes* ERA (East River isolate A; ERA), which poses a relatively greater opportunity for adaptive improvement [7,26]. Thus, we expected that the treatment populations evolving on PP and ERA hosts under large population sizes would experience positive selection (of differing strengths) to fix adaptive mutations. In addition, our experiment subjected some virus lineages to extreme population bottlenecking which should cause drift to overwhelm selection, allowing random fixation of non-lethal mutations of moderate effect [29,35]. Phage evolution occurred strictly through the ~4 generations per day as plaques grew on bacterial lawns [7,29,30] (see Methods); no phage infections occurred in liquid culture.

**Figure 2:**
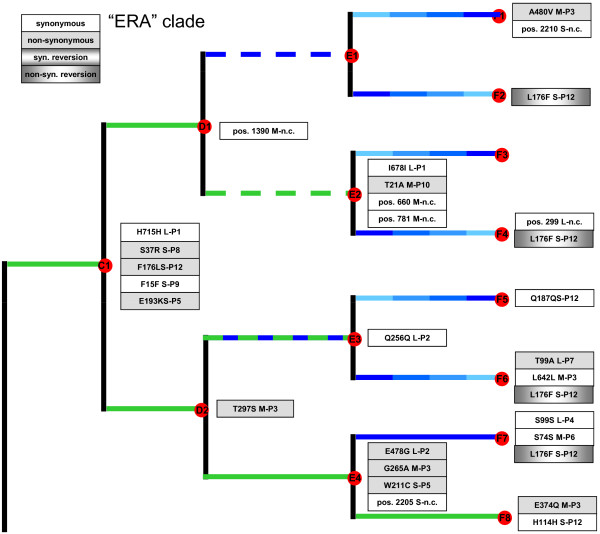
**Molecular changes observed in the experimental clade more often experiencing infection on *P. pseudoalcaligenes *ERA (“ERA clade”).** Changes in coding regions list the affected protein amino-acid substitution, RNA segment (L, M or S) and gene; changes in non-coding (n.c.) regions list the base position and segment. Mutations are relative to those observed in the immediate predecessor clone

**Figure 3:**
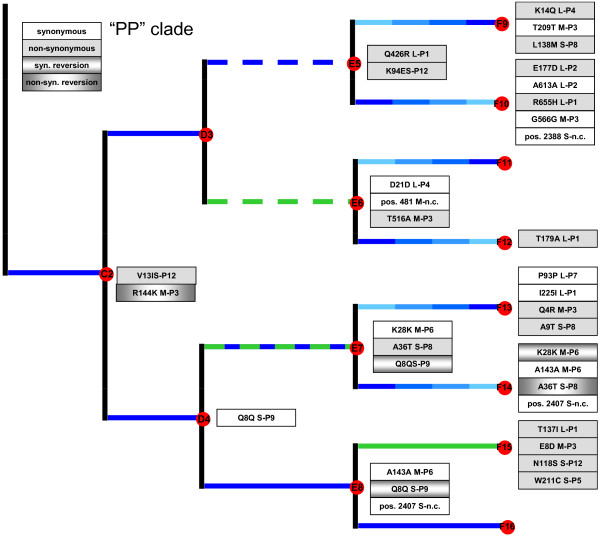
**Molecular changes observed in the experimental clade more often experiencing infection on *P. phaseolicola *(“PP clade”).** Changes in coding regions list the affected protein amino-acid substitution, RNA segment (L, M or S) and gene; changes in non-coding (n.c.) regions list the base position and segment. Mutations are relative to those observed in the immediate predecessor clone

We used whole-genome sequencing to identify molecular changes that occurred in the experiment. Figures 2 and 3 summarize the observed mutations, and the time points and treatment regimes where they were identified; the indicated mutations are those separating a clone from the sequenced predecessor clone.

We founded the experiment with a wild type ϕ6 ancestor (i.e., strain A1; Figure 1); after 50 phage generations on PP we observed that this starting lineage contained no fixed mutations separating it from strain A1. Because this lineage was designed to give rise to a bifurcation where one of the two derived lineages would be propagated on host ERA (Figure 1), we chose a spontaneous host-range mutant able to grow on both PP and ERA, from the initial 50-gen. lineage. This mutant, denoted clone B1, contained a non-synonymous mutation (K144R) in gene P3 of the M segment, the locus for the host attachment protein [32,36]. We note that this P3 mutation differs from the nine known non-synonymous substitutions in gene P3 which allow phage ϕ6 to infect the ERA host [36].

By the end of the study, each of the 16 clones at the tips of the tree differed from the ancestor (clone A1) by 4 to 13 substitutions (mean = 8.2 ± 2.7 s.d.), excluding reversions (Figures 2 and 3). Overall, we observed a total of 65 substitutions (including reversions) at 54 sites across the 3 genomic segments, equivalent to ~0.004% of the sequenced genome (12,478 bp).

**Figure 4:**
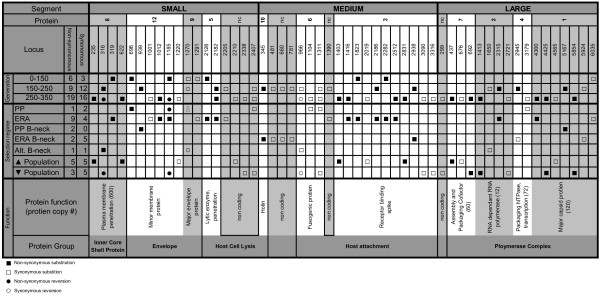
Molecular changes, in light of segment location, treatment regime and protein function

Figure 4 summarizes all molecular changes stratified by total evolutionary time (virus generations) and selection regimes arrayed along genomic segments and annotated by putative protein function. The observed substitutions were distributed disproportionately among the three segments. Changes occurred on average every 375 bp in the L segment, every 193 bp in the M segment, and every 109 bp in the S segment, which is significantly different from uniform distribution across the genome (chi-square test, p <0.05). This finding suggested that the size of a segment was negatively correlated with its propensity to change under the environmental challenges, most likely owing to the functional properties of genes residing on each segment and their tolerance for genetic change (Figure 4). We speculate that S segment genes were more often the target of selection under the treatment conditions we imposed. Although S is a relatively smaller molecular target, it holds a greater variety of functions, and might have changed more often because the numerous selection conditions targeted a wide variety of functions. A related issue is that the L segment should be generally less prone to change, given the expected strong selection to maintain existing polymerase function.

Figure 4 also shows calculations of the ratios of non-synonymous substitutions (K_a_) to synonymous substitutions (K_s_) according to treatment regime. The strongest signal of positive selection was for virus lineages evolved for 450 combined generations at large population size on novel host ERA: K_a_/K_s_ = 9/4. In contrast, the lineages evolved for 500 combined generations at large population size on the typical host PP underwent relatively little molecular change and fewer amino-acid replacements: K_a_/K_s_ = 1/2, consistent with the idea that the phage was already well-adapted to the typical host. The bottlenecking treatments that each occurred for 200 generations showed variable results, but were not significantly different from each other by pairwise Fisher’s exact test (p > 0.05): PP bottlenecks produced 2 non-synonymous and 0 synonymous mutations, ERA bottlenecks produced 2 non-synonymous and 5 synonymous mutations, and alternating-host bottlenecks produced 1 non-synonymous and 1 synonymous mutation. The treatment that imposed increasing population sizes occurred for 600 combined generations and produced 5 non-synonymous and 5 synonymous mutations, whereas the identical regime imposing decreasing population sizes produced 3 non-synonymous and 5 synonymous mutations (Fisher’s exact test, p > 0.66).

### Reversion mutations

We observed that identical reversion mutations sometimes occurred in lineages evolved independently. One case involved evolution under host switching, whereas the other did not. The host-switching case was a substitution on segment S in gene P12 (Figure 2), which encodes an assembly protein used in viral membrane morphogenesis [37]. This non-synonymous substitution was a thymine to cytosine transition (F176L), observed in clone C1 isolated following the first experimental bifurcation, in the ‘host shift’ lineage that had evolved for 50 generations on novel host ERA (Figure 2). The P12 substitution persisted in descendent lineages propagated at constant large population sizes on ERA, and in lineages bottlenecked at constant small population sizes regardless of host (PP, ERA, alternating PP/ERA). However, in all cases where descendent lineages were grown at constant large or initially large population sizes on PP, the P12 mutation underwent a reversion: L176F (Figure 2). The combined influences of host type and population size (e.g., efficiency of selection) on forward/back mutation suggested that these alleles governed host-specific growth performance. Below we describe additional evolution experiments that further examined this finding (see *Evolution under host switching*).

The reversion unrelated to host-switching was also a mutation on the S segment: 1270 A → G in gene P9, which codes for a membrane protein essential for virion membrane formation [38,39]. The mutation occurred in a lineage experiencing selection at constant large population sizes on PP, which gave rise to one of the third set of experimental bifurcations (Figure 3). The substitution then reverted in the two immediately descendent lineages, which were either bottlenecked at constant small population sizes on alternating hosts, or further evolved at constant large population sizes on PP. One possibility is that the mutation in gene P9 is antagonistic for growth on host ERA, explaining why it was selected against in the alternating PP/ERA bottlenecking; although the bottlenecking method caused drift to overwhelm selection, some positive selection necessarily occurred during plaque formation (see *Accounting for low rates of molecular change* for detailed explanation). The reversion that occurred under continued PP-selection at large population size suggests that the mutation may also be antagonistic with additional mutations fostering growth on the normal host. In particular, the reversion was observed alongside two synonymous mutations on segments M and S (Figure 3); although these were synonymous mutations they could potentially affect membrane formation properties of protein P9 and/or protein-protein interactions with other viral proteins that affect performance on the PP host. This suggestion is highly speculative, however, and further experiments are warranted to confirm the idea.

### Accounting for low rates of molecular change

The spontaneous mutation rate in phage ϕ6 is estimated to be 2 × 10^-6^ mutations/base/round of replication [40]; this rate provides the genetic variation potentially useful for adaptation, but it is relatively low compared to the typical error-prone replication rates associated with RNA viruses [6,41]. One possible explanation is that the inferred ‘stamping machine’ model of RNA segment replication in phage ϕ6 should lead to fewer mutations generated per infected cell, compared to a geometric mode of replication occurring in other RNA viruses [40]. Regardless of the mechanistic explanation, one key result from our study was that molecular evolutionary changes were rather modest after 350 viral generations (sum total of 2750 generations, adding together the generations occurring on all of the branches in the tree; Figure 1); clones isolated at the tree tips sometimes contained only 4 substitutions separating them from the wildtype ancestor. Although phage ϕ6 experiments on the order of 50 to 300 generations can produce profound phenotypic changes under strong directional selection [30,35,42], previous work and the current study suggest that few underlying genetic substitutions may fix during such timeframes [7] perhaps owing to strong clonal interference among mutations of similar magnitude in their beneficial effects.

Severe bottlenecking increases mutational load in phage ϕ6 populations through effects of drift [29,35]. However, we estimate that 20 days of consecutive bottlenecking should be required to fix roughly one mutation, on average. The logic is the following. The estimated genomic mutation rate in phage ϕ6 is gauged to be 0.067 deleterious mutations per generation [43], causing one mutation on average to fix in a lineage after 20 bottlenecks (i.e., 0.067 × 20 bottleneck events ≈ 1.3), where the majority of spontaneous mutations are assumed to be deleterious. Thus, the 20-day bottlenecking treatments may have caused fitness declines, but they were unexpected to cause large numbers of mutations to accumulate. In addition, to impose the conditions which fostered drift over selection, we used the reliable method of randomly choosing a single plaque (extreme bottleneck of *N* = 1) to propagate the evolving virus lineage [29,30,35]. However, this method allows for considerable within-plaque selection; despite the tight bottleneck, the ~4 generations of virus growth needed to produce a visible plaque allow for positive selection occurring during this process [35]. For these reasons, our bottlenecking method was unlikely to cause large numbers of fixed molecular substitutions.

In the future, similar studies employing phage ϕ6 could use a mutagen to achieve higher mutation rates, and relatively greater occurrence of substitutions per unit time. However, we caution that while the mutation rates of certain viruses can be manipulated by mutagens, the resulting artificial mutant spectrum compromises inferences drawn from a ‘known phylogeny’ experiment. For instance, mutagenic deaminating agents cause substitution biases (G → A, C → T) that cannot be accurately modeled with reversible substitution models [44]. Two solutions for avoiding insufficient change in known-phylogeny experiments with viruses are to allow evolution to proceed for a relatively long time, and to evolve the phylogeny under conditions of continuous-growth (e.g. in a chemostat) where very many generations can occur in a single day.

### Fitness evolution: growth on P. phaseolicola

For the ancestor and sequenced clones, we measured a phenotypic trait that was often a direct target of selection in our study: fitness (*W*) on the PP host. We observed that all viruses retained the ability to infect the original PP host even when evolved solely on ERA; in contrast, Duffy et al. [7] showed that strict ERA selection can sometimes lead to fixation of a non-synonymous mutation in P3 (not observed in the current study) that prevents phage ϕ6 entry into PP host cells. Thus, for all 32 sequenced viruses we conducted fitness assays on PP against a genetically-marked common competitor that contained a host-range mutation on segment M, and an inserted X-gal mutation on segment L (see Methods). Assays were performed with six-fold replication, yielding 192 total measures (32 clones × 6 replicates). These measures were log-transformed to improve normality.

Because PP is the typical lab host for phage ϕ6, the *a priori* prediction was that viruses evolved at large population sizes on this host should show equal or higher fitness relative to the ancestor (clone A1). Table 1 shows mean ln*W* for each virus on PP. The mean ln*W* of the ancestral clone A1 was determined to be significantly greater than 0.0 (*t*-test with *t* = 11.71, df = 5, *P* <0.001), indicating that the ancestor was more fit on PP than the common competitor. We then conducted independent *t*-tests to gauge whether the mean ln*W* of a test virus differed from that of the ancestor; because these tests involved 31 comparisons versus the ancestor dataset we conservatively gauged significance using a Bonferroni correction of α = 0.0016 (i.e., 0.05 / 31). We observed that fitness on PP statistically differed from the ancestor in a majority of these comparisons (19 of 31 tests), and most of these outcomes showed fitness significantly lower than the ancestor (18 of 19 tests). Consistent with our prediction, close inspection of these data and the experimental design revealed that all clones which had recently experienced constant selection on PP at large population size (i.e., clones B1, C2, D3, D4, E8, F7, F16) did not suffer a decrease in fitness on PP (cf. Figure 1, Table 1). In contrast, 4 of 6 clones which had recently undergone constant selection on ERA at large size (i.e., clones C1, D1, D2, E4) suffered a decline in fitness on PP. These data suggested that selection for fitness improvement on ERA tended to trade off with performance on PP see also [26].

**Table 1:** **Fitness of evolved viruses relative to the ancestral and predecessor strains, on the typical host *P. phaseolicola *, and the novel host *P. pseudoalcaligenes *ERA**

**Clone**	**Fitness on *PP***^**1**^	**Relative to A1**^**2**^	**Relative to predecessor**^**3**^	**Fitness on ERA**^**1**^	**Relative to B1**^**4**^	**Relative to predecessor**^**3**^
A1 (anc)	0.298 (0.062)	n.a.	n.a.	---	n.a.	n.a.
B1	0.180 (0.226)			−1.373 (0.262)	n.a.	n.a.
C1	−0.630 (0.219)	↓	↓	−0.437 (0.221)	↑	↑
C2	0.121 (0.168)			---	---	---
D1	−0.875 (0.083)	↓		−0.277 (0.159)	↑	
D2	−0.697 (0.236)	↓		−0.527 (0.316)	↑	
D3	0.272 (0.167)			---	---	---
D4	0.327 (0.069)		↑	---	---	---
E1	−0.935 (0.140)	↓		−0.432 (0.097)	↑	
E2	−0.855 (0.123)	↓		−0.256 (0.104)	↑	
E3	−0.651 (0.199)	↓		−0.395 (0.222)	↑	
E4	−0.640 (0.170)	↓		0.075 (0.275)	↑	↑
E5	−0.583 (0.185)	↓	↓	---	---	---
E6	0.287 (0.065)			−0.379 (0.237)	↑	---
E7	−0.490 (0.122)	↓	↓	---	---	---
E8	0.362 (0.228)			---	---	---
F1	−0.480 (0.209)	↓	↑	---	---	---
F2	−0.219 (0.264)	↓	↑	0.005 (0.265)	↑	↑
F3	−0.694 (0.167)	↓		−0.264 (0.140)	↑	
F4	0.142 (0.041)	↑	↑	−0.242 (0.163)	↑	
F5	−0.550 (0.278)	↓		−0.045 (0.182)	↑	↑
F6	−0.401 (0.078)	↓	↑	---	---	---
F7	0.387 (0.108)		↑	0.510 (0.242)	↑	↑
F8	0.101 (0.157)		↑	0.698 (0.122)	↑	↑
F9	−0.058 (0.114)	↓	↑	---	---	---
F10	−0.066 (0.050)	↓	↑	---	---	---
F11	0.283 (0.181)			−0.332 (0.258)	↑	
F12	0.287 (0.101)			−0.222 (0.185)	↑	
F13	−0.048 (0.168)	↓	↑	---	---	---
F14	−0.845 (0.199)	↓	↓	---	---	---
F15	0.494 (0.178)			1.028 (0.148)	↑	---
F16	0.445 (0.117)			---	---	---

^**1**^**Values are means (std. dev.) of six log fitness estimates; ↑ and ↓ indicate significantly higher and lower fitness relative to ancestor or to immediate predecessor clone; open cells indicate no statistical difference; --- indicates test could not be performed.**

^**2**^**Tested relative to Bonferroni corrected probability of 0.0016.**

^**3**^**Tested relative to Bonferroni corrected probability of 0.025.**

^**4**^**Tested relative to Bonferroni corrected probability of 0.003.**

Fitness declines on PP were also observed for clones which were bottlenecked on either PP, ERA or alternating PP/ERA, and whose immediate predecessor clone showed significantly lower fitness on PP (i.e., clones E1, E2, E3); these data were consistent with predicted effects of bottlenecking that cause drift to overwhelm positive selection [29], preventing the lineages from regaining fitness. For a detailed summary of the inferred effects of population size on observed evolution of clone fitness on PP, see the Additional file 1.

**Figure 5:**
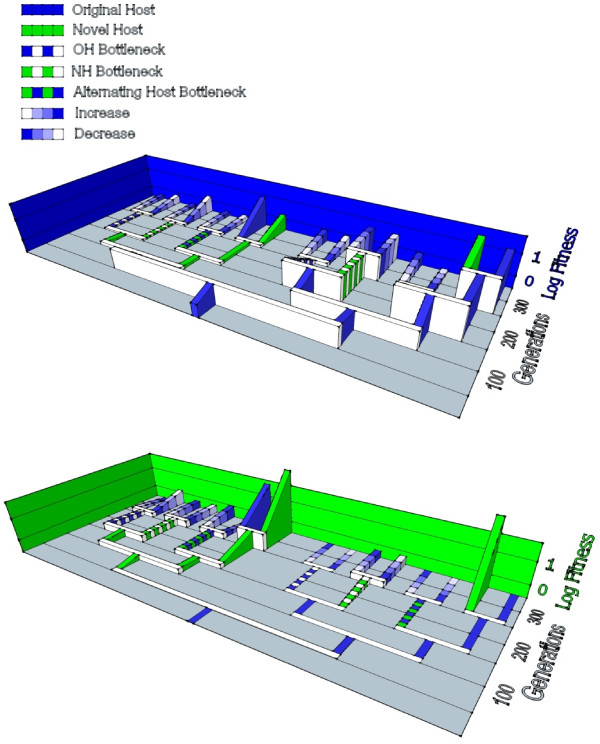
**Phenotypic evolution through time, in light of the imposed experimental design.** Values are mean log fitness of sequenced clones on (**A**) *P. phaseolicola* (PP) and on (**B**) *P. pseudoalcaligenes* (ERA). See Table 1 for numerical values and statistics

Figure 5A depicts the measured fitness changes on PP, in relation to the bifurcating demes and two major clades created in our study. It is evident that fitness on PP tended to be much lower for lineages in the clade where early evolution occurred on ERA, whereas it was higher for the lineages in the clade that mostly experienced PP. Again, this observation suggested that evolution on ERA tended to trade off with performance on PP.

### Fitness evolution: growth on P. pseudoalcaligenes ERA

We also determined how the fitness of viruses on ERA changed in our study. Clone B1 was a direct descendent of the ancestor, and contained a spontaneous host-range mutation (K144R) in gene P3 on the M segment, allowing infection of novel host ERA (Figure 1). We sought to examine fitness on ERA for the 30 clones derived from clone B1, but assays on ERA were not possible for a subset of these viruses because they were never directly exposed to ERA and therefore had experienced a reverse mutation (R144K) preventing infection of ERA (i.e., *W* = 0.0, ln*W* undefined). Thus, fitness assays against the common competitor on ERA were conducted for only 18 clones, yielding a total of 108 measures (18 clones × 6 replicates), which were log-transformed to improve normality.

Our *a priori* prediction was that evolution on ERA at large population size should lead to strong performance on this host. Table 1 shows mean ln*W* for the 18 viruses assayed on ERA. The mean ln*W* of clone B1 was determined to be significantly lower than 0.0 (*t*-test with *t* = −12.83, df = 5, *P* <0.001), indicating that clone B1 was less fit on ERA than the common competitor. Independent *t*-tests gauged whether mean ln*W* of a test virus differed from that of clone B1; because these tests involved 17 comparisons versus B1 we employed a Bonferroni correction of α = 0.003 (i.e., 0.05 / 17). We observed that fitness on ERA was significantly greater than that of clone B1 in every comparison. This outcome was consistent with the predicted improvement on ERA for clones recently experiencing evolution on ERA at large population size (i.e., clones C1, D1, D2, E4, F8, F15). Interestingly, clones E1 thru E3 were direct descendents of strong performers on ERA and maintained high fitness on this host despite bottlenecking. The E1 clone, by chance did not experience any mutations from its D1 ancestor. For E2 and E3, the result suggested that ERA performance was less affected by drift occurring during the 20-day bottlenecks, compared with typical debilitating effects of 20-day bottlenecking on PP fitness [29,35]; related to the above suggestion, one possibility is that within-plaque growth/selection more easily counters the effects of drift when phage ϕ6 is bottlenecked on ERA, compared to identical propagation on PP. The phenomenon may also explain why clone E6 showed high fitness on ERA despite being subjected to bottlenecks on this host. For additional discussion of ERA performance shown by individual clones, see the Additional file 1. Figure 5B summarizes the fitness values observed on ERA, in relation to our experimental design. It is obvious that the clade containing lineages mostly selected on ERA tended to show high fitness values on ERA.

### Fitness evolution relative to immediate predecessors

To examine fitness changes on selected and unselected hosts over evolutionary time, we compared the phenotype of a virus clone to that of its immediate predecessor clone: the virus used to initiate a bifurcation that gave rise to the descendant clone. This effort yielded up to 31 statistical comparisons for each of the three measured traits, where we employed a Bonferroni correction of α = 0.025 for determining significant differences in *t*-tests where two clones were tested relative to their common progenitor.

We observed that mean ln*W* of a virus clone on the typical host PP did not always statistically differ from that of its predecessor virus (Table 1). Fitness on PP tended to be equal for clones isolated from lineages successively evolved on PP at constant large size (i.e., clones A1-B1-C2-D4-E8-F16; Figure 1); a linear regression showed that this trajectory of ln*W* through time had positive slope but did not differ from zero (*m* = 6.7 × 10^−4^, *P* = 0.10). However, fitness on ERA showed a significantly positive increase through time (linear regression: *m* = 6.1 × 10^−3^, *P* = 0.01) for clones from lineages successively evolved on ERA (i.e., B1-C1-D2-E4-F8; Figure 1). We found some evidence that the viruses constantly evolved on ERA suffered a tradeoff in performance on PP; the regression of differential performance across hosts (ln*W*_ERA_ – ln*W*_PP_) versus time was a decreasing function, but did not differ statistically from zero (*m* = −1.2 × 10^−3^, *P* = 0.67). A similar analysis was not possible for differential performance of PP-evolved viruses across hosts, because most of these clones could not infect ERA (Table 1). Additional discussion of individual clone performance on PP and/or ERA relative to the immediate predecessor is contained in the Additional file 1.

### Limitations to inferring genotype-phenotype associations

We note one important caveat in interpreting the phenotypic consequences of the molecular substitutions stems from our acquisition of clones from each population to conduct whole-genome sequencing. At the end of each treatment period, as detailed in the Methods, a single clone was chosen and this clone was then subjected to sequencing. For example, we began a selective regime with a clone whose sequence was known, the clone was expanded into a population and that population underwent a particular selective regime for a period of time. Subsequently, an individual clone was selected at random from the population, sequenced and measured phenotypically. We then inferred from the changes separating the starting clone and the endpoint clone how the virus population responded to selection. Through this method, there was a chance that the clone we chose was not representative of the parent population. Thus, the molecular changes we observed were not necessarily reflective of measurable performance changes in the parent population; see also [35] for related discussion. However, given the overall low number of observed changes in the experimental evolution, we are confident that a discrepancy between clone-level changes and majority alleles in the parent population should occur with low probability, in turn suggesting that the above-described associations should be generally robust interpretations.

### Evolution under host switching

The importance of the P3 protein in host switching events in phage ϕ6 has been previously established [36,45]. This study confirmed this observation, but additionally brought to light the importance of the P12 protein in host switching events. This non-structural protein controls the liberation of mature ϕ6 particles from the host cytoplasmic membrane, but is not incorporated into the virion [46]. Like many eukaryotic viruses, *Cystoviridae* have envelopes comprised of both viral proteins and host lipids. Different hosts may have different lipid constituents which are contributed to the viral envelope and thus may require slightly altered P12 proteins for efficient envelope assembly. We have already shown that phage ϕ6 maturation in ERA affects fitness when the virion infects PP, and *vice versa*[25,36]. We assume this epigenetic effect is mediated by the lipids taken from these very different hosts, and we speculate that our current results may relate to the importance of host lipid incorporation for multiple host-use in phage ϕ6.

As described above, we observed that non-synonymous mutation F176L in gene P12 on the S segment was associated with a host switch to the novel host ERA. This mutation seemed to revert when a virus lineage was subsequently allowed to evolve on the typical host PP, as long as population size was constantly large or decreased from large size to small size (Figure 2). We hypothesized that the F176L mutation was beneficial for ERA infection, but that the L176F reversion was beneficial for growth on PP.

To confirm the hypothesized functional significance of the allelic switch for growth on PP and ERA, we conducted follow-up experimental evolution to test whether population size and host type affected allele fixation in test populations. The design for this experiment is shown in Additional file 1. We isolated a single plaque-purified copy of clone C1 that had evolved on ERA at large population sizes; sequencing confirmed that this clone contained the F176L mutation presumably beneficial for ERA infection. This clone was used to found four additional virus lineages that were evolved on PP for 20 days (100 generations) at population bottlenecks of either 10, 100, 10^3^, or 10^4^ pfu (Additional file 1). After the evolution experiment, we used targeted consensus sequencing of each test population to examine changes in the P12 gene.

We observed that the L176F reversion was the dominant allele (>92%) in the experimental populations evolved at population sizes of 10^3^ and 10^4^ pfu. In contrast, the allelic reversion did not fix (<8%; limit of detection) in the test populations evolved at bottleneck sizes of 10 and 100 pfu. We then conducted a repeat of this evolution experiment in a separate block (Additional file 1), and observed qualitatively identical results. These data strongly supported the idea that the non-synonymous P12 mutation was beneficial for growth on ERA and deleterious for performance on PP, whereas the reversion produced the opposite function. We concluded that population sizes of 10^3^ and larger were sufficient for selection to efficiently drive the reversion to high frequencies within 100 generations, when viruses were cultured on the original PP host see also [30].

Why does fixation or dominance of reversion mutation happen at some population sizes and not others? Current literature emphasizes that waiting time for selective sweeps does not necessarily explain what happens in clonal microbial populations. Rather, there is more likely genetic variation present at any one time and selection draws upon this variation to produce change; there is thus an importance of clonal interference among variants of similar magnitude. In our lineages where population size started large and became small, we observed that the reversion fixed (or nearly fixed). Likely such lineages of initially large size already had the needed revertant present within their existing variation, and it was able to spread through time even though population size diminished. In contrast, the lineages where population size started small and increased had to wait for the revertant to appear, because the initially small size made it unlikely that the needed genotype was initially present. The result was that the revertant never fixed because enough time did not elapse at large size for that revertant to exist within the standing genetic variation.

But when population size was sufficiently large enough for a lineage subsequently evolved on PP, the beneficial revertant was able to spontaneously arise and fix in the population. Because ERA and PP are distantly related [27,28], one possible explanation is that the substitution in the P12 assembly protein enables more effective incorporation of ERA membrane lipids into the virus envelope at the expense of effectively incorporating PP membrane lipids.

### Phylogenetic reconstruction

**Figure 6:**
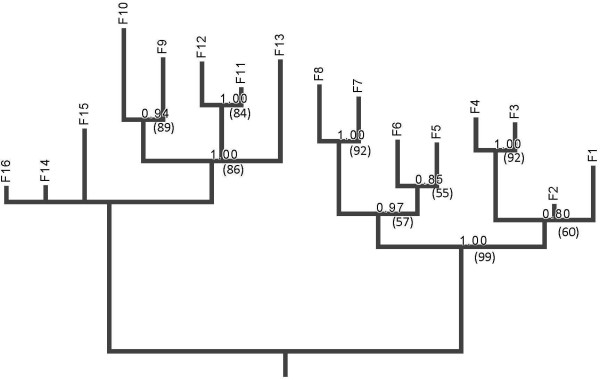
**Inferred topology using only aligned sequences from tips (clones F1 thru F16) of the tree.** Maximum parsimony, maximum likelihood, and Bayesian methods all yielded the same inferred tree (see text for details). MrBayes provided estimates of branch lengths. Numbers at nodes indicate marginal posterior probabilities, and bootstrap percentages in parentheses

The bifurcating-deme structure we imposed in our experiment afforded the possibility to test whether phylogenetic methods could successfully reconstruct the tree topology using genetic data alone. All methods returned the same phylogeny with similar assessment of uncertainty by bootstrap or posterior probability (Figure 6). The lineage containing descendants of C1 (“ERA” clade; Figure 2), were reconstructed with no false positive clades and one false negative clade (F1 and F2 were not recognized as a clade). The reconstructions for the descendants of C2 (“PP” clade; Figure 3) were more mixed with several false negatives and one false positive. The false negatives involved failure to identify the clades descended from D2 while the false positive involved placing the F13 line together with F9-F12, creating an erroneous clade of {F9, F10, F11, F12, F13}. The false negatives, that is, the failure to resolve certain branches, is due to the small number of polymorphic sites within our dataset. Purifying and directional selection over linked loci seems to have greatly reduced the phylogenetically informative sites in our study. Some of the problems with the reconstruction can be traced to particular mutational history. As noted, the clade {F13, F14, F15, F16} is unresolved in the estimated tree. The clade descendant from D4 is delineated by Q8Q (1270 A→G) mutation in the P9 gene on segment S. However, in both E7 and E8, direct descendants of D4, there was a reversal synonymous Q8Q (1270 G→A) mutation that erased the evolutionary history (Figure 3). A parallel mutation of A143A (1311 A→G) in M-P6 gene in both the E8 and F14 lineages seems to be the main signature that caused F13 to erroneously fall into the clade that contains F9, F10, F11, and F12. Finally, we used maximum likelihood ancestral state estimation in PAUP to reconstruct the ancestral sequences. All ancestral states were correctly reconstructed with the exception of two sites. The first site was the Q8Q(1270 A→G) site, which as described, reverted back to the ancestral state in two descendent lineages, in effect completely hiding the mutation in the terminal lineages. No algorithm is expected to reconstruct such exact reversions. The second site is the highly variable F176L mutation in P12 gene of segment S discussed above, which showed parallel changes in four terminal lineages. The terminal mutations were all C to T changes in the terminal lineages, which are reversions to state T of the common ancestor to all lineages, following an early T to C change in the “ERA” clade. Therefore, the true ancestral state was C but was incorrectly estimated to the T state, reflecting the shared T state in the “PP” clade as well as the T’s in the other terminal lineage of the “ERA” clade. The high degree of parallel terminal mutations is highly unlikely by standard probabilistic models, as well as by the parsimony criterion and the ancestral states of such singular events are not expected to be estimated correctly. In sum, complex evolutionary history, especially with selective pressures that can cause parallel or reversal of specific loci, can cause difficulties with phylogenetic reconstruction even when the whole sequenced genome is available.

Regarding use of our dataset to investigate how environmental complexities (e.g., variable selection along branches) impact the ability for phylogenetic methods to accurately infer historical relationships, we observed that none of the investigated methods could accurately reconstruct the true phylogeny. This result is similar to the observations of Bull et al. [11] in a previous known phylogeny study. In the prior experiment, the failure was due to the effects of convergence of the phylogenetic topography. However, in the current study we showed that the failure could occur due to a paucity of observed molecular changes. For example, two of the sixteen endpoint lineages presented exactly the same genetic sequence, rendering it impossible to distinguish them, regardless of the method employed. Thus, we could not effectively assess how variable selection impacted the usefulness of various methods of phylogenetic reconstruction.

Overall, the results of our dataset were not useful for evaluating the relative effectiveness of the various methods due to the paucity of changes in some lineages. However, when examining how well the methods performed on the clade produced from the initial switch to ERA, we observed that the methods were more accurate in inferring the actual phylogeny; in particular, ML again proved to be the most effective method for accurately regenerating the actual clade.

## Conclusions

Phylogenetic methods can often fail to resolve deep relationships with saturated molecular data; here we showed a similar difficulty in using the methods to resolve the simple bifurcating design implemented in this ~350 generation known phylogeny experiment. Using the natural mutation rate of this RNA virus and environments similar to the selective pressures and population size changes experienced by many viruses in nature, we were able to demonstrate several aspects of viral evolution that can confound the usefulness of phylogenetic algorithms: few informative sites, and parallel and reversion mutations. Because these complications will often occur when tracking the evolution of emerging viruses, our work implies that phylogenetic trees encompassing molecular epidemiology over short timescales may tend to be inaccurate. Specific to the study system, we identified that the protein-coding gene P12 on the S segment of phage ϕ6 is important for host-use selection in the virus. Our study demonstrated that experimental evolution involving a large number of increasingly diverged lineages is a useful tool for testing evolutionary theory (accuracy of phylogenetic algorithms), as well as for discovering novel traits (i.e., novel functions of P12) in evolving populations that more narrowly-focused studies would omit.

## Methods

### Strains and culture conditions

The ancestor (‘wild type’) was a plaque-purified clone of phage ϕ6 (strain #21781-B1, American Type Culture Collection, Bethesda, MD), a member of family *Cystoviridae*: tri-partite dsRNA phage with lipid envelopes [31,32]. Phage ϕ6 is typically cultured on *P*. *phaseolicola* strain HB10Y (ATCC #21781). Mutations in gene P3 (attachment protein, M segment) allow phage ϕ6 to infect novel hosts [36], including *P. pseudoalcaligenes* ERA (provided by L. Mindich, Public Health Research Institute, Newark, NJ).

We grew bacteria at 25°C in LC medium: Luria broth (10 g NaCl, 10 g Bacto® tryptone, and 5 g Bacto® yeast extract per liter) at pH 7.5 [26]. We initiated a culture by growing a single colony overnight in 10 ml LC medium with 120 rpm shaking, to attain stationary-phase density (PP: ~4 × 10^9^ cells ml^−1^; ERA: ~5 × 10^10^ cells ml^−1^). We stored bacterial stocks at −80°C in a 4:6 glycerol/LC (v/v) solution.

We grew phage by mixing up to ~10^4^ virus plaque-forming units (pfu) with ~8 × 10^8^ stationary-phase bacterial cells in 3 ml of 0.7% top agar, overlaid on a 1.5% agar plate. After 24-hour incubation at 25°C, the viruses formed visible plaques (holes) in the bacterial lawn growing in the agar overlay. The initial phage to bacteria ratio caused the vast majority of infections to be clonal (i.e., each virus infected an individual cell). Thus, we assumed a single virus initiated a plaque, which contained ~10^10^ pfu resulting from ~5 generations of virus growth; this estimate assumes a burst size of ~100 particles per infected cell in each generation (i.e., 100^5^ = 10^10^ pfu) [24]. When plating inocula of ~10^3^ – 10^4^ pfu, this produced a ‘lacey lawn’ characterized by considerable overlap among the resulting ~10^3^ – 10^4^ plaques during late stages of growth on agar. Plaque overlap can cause phage to locally outnumber bacteria, permitting virus co-infection of the same cell and segment reassortment (genetic exchange) between phage genotypes known to occur in nature [47,48]. However, this method produces only a tiny minority subpopulation of reassortants, unlike experiments where ‘infective centers’ (cells with multiple pre-adsorbed phage) are plated to promote high levels of co-infection throughout plaque growth [24,42]. We prepared virus lysates by harvesting plaques into LC broth, followed by 10 min centrifugation at 3000 rpm; we filtered (0.22 μm, Durapore®; Millipore, Bedford, MA) supernatant to remove bacteria. We serially transferred a lineage by diluting the lysate to repeat the process described above. In all experiments, we grew phage on naïve (non co-evolved) bacteria freshly prepared from frozen stock, to prevent the possibility that bacteria would evolve resistance to phage attack. We stored lysates and virus clones (single plaques isolated from top agar) at −20^o^C in 4:6 glycerol/LC.

### Experimental evolution conditions

We used phage ϕ6 inoculum containing ~10^3^ pfu to found a single lineage, evolved for 10 days (50 phage generations) of serial transfer on *P. phaseolicola* (hereafter ‘PP’) lawns. This evolving lineage fluctuated between the imposed transfer size of ~10^3^ individuals (i.e., pfu placed in the agar at each serial transfer) and a maximum of ~10^13^ individuals (i.e., 10^3^ resulting plaques multiplied by 10^10^ pfu per plaque). Thus, each serial transfer imposed a bottleneck in the number of individuals that resulted from the unrestricted growth on the agar plates. After 10 days we bifurcated the lineage to create two new lineages: one evolved on novel host *P. pseudoalcaligenes* ERA (hereafter ‘ERA’) and one that continued evolution on PP (Figure 1). To do so, the day-10 lineage was diluted onto an ERA lawn to obtain distinct (non-overlapping) plaques, containing virus particles capable of infecting both PP and ERA. A sample from this plaque founded the two lineages that evolved separately under the above conditions, for 10 additional days. We stored a copy of this clone (and all others giving rise to bifurcation events) in the freezer for later analysis. We used this general protocol with variations in the bottleneck sizes at each serial transfer to create our 16-tip bifurcated tree (Figure 1).

The study contained five other selection treatments. Two treatments matched the PP and ERA passages, except that each day the evolving virus lineage experienced a bottleneck size of *N* = 1, where a single plaque was isolated at random and used to propagate the population following described methods [29]. This bottleneck caused drift to overwhelm the 5 generations of positive selection occurring during plaque formation, allowing non-lethal mutations to fix in the evolving lineage [29,30,35]. A similar treatment imposed bottlenecks of *N* = 1, but using daily alternating infections of PP and ERA hosts. Last, two additional treatments imposed differing bottlenecks (i.e., *N* = 10, 10^2^, 10^3^, and 10^4^) that increased or decreased in size. For each of these transfers, we obtained plaques from the entire plate and titered them to the appropriate size; thus, the next generation represented a random subset of plaque clones from the previous generation. We imposed treatments for either 10 or 20 days (Figure 1). Whereas experimental evolution studies often contain replicated treatment populations founded by a common ancestor (i.e., polytomy: node with more than two immediate descending branches) [7,24,26,49], our study purposefully included a bifurcating deme design consistent with prior known phylogeny studies [10,12], and replicated treatments nested within the tree.

Total duration of the experiment was 350 generations from the root of the tree to the tips; the entire tree represented 2750 generations of evolution. In addition to the clone samples taken at the nodes of the tree, we obtained single clones at the tips. This scheme yielded 32 clones for genome sequencing (16 tips + 15 nodes + 1 ancestor). Also, we isolated population samples every 5 days of evolution and stored these in the freezer for future analyses.

### Sequencing

We used a virus clone to obtain a high-titer lysate, followed by genomic RNA extraction (QiaAMP viral RNA extraction kit; Qiagen Inc., Valencia, CA) and conversion to cDNA using RT-PCR with Superscript polymerase and random hexamer primers (Invitrogen, Carlsbad, CA). We used standard PCR methods to amplify 93.2% of the genome excluding the single-stranded ends of each segment [7], and purified PCR reactions for sequencing using ExoSAP-It (US Biological, Swampscott, MA). The University of Pennsylvania DNA Sequencing Facility Sanger performed sequencing using standard methods; we ran sufficient reactions to ensure double-coverage of every nucleotide in each of the 32 nearly whole-genome sequences. All sequences are available through Genbank (accession numbers: JX481790 – JX481885).

### Phylogenetic analyses

We performed phylogenetic reconstructions using maximum parsimony (MP) and maximum likelihood (ML) in PAUP* [50], and Bayesian estimate using MrBayes [51]. We aligned L, M, and S segments of the genomes into a concatenated dataset. Multiple sequence alignment was straightforward as no indels were observed in the sequences. For MP, we exhaustively searched the tree space by the branch and bound method, and we used bootstrap re-sampling to assess clade replication using 500 replicates. For the probability models, we first used MrBayes with GTR model and Gamma + Inv and examined the posterior parameter estimates. The estimated gamma parameter and proportion of invariant sites suggested two nearly equally probable models: a model with no invariant sites and high rate variation and a model with high proportion of invariant sites (0.97) and no rate variation. Given the low level of variant sites in our dataset, for the remaining analysis we used GTR as base model and invariant proportion of 0.97 for both the ML and Bayesian analyses. ML analysis was carried out for 500 bootstrap replications using TBR heuristic search and MrBayes analysis was carried out to 200,000 samples with 50% burn-in; all diagnostic statistics indicated sufficient convergence by 120,000 generations.

### Fitness assays

Using published methods [29] we assayed fitness on PP, as well as on ERA if the test phage contained a host-range mutation. We measured fitness relative to common competitor phage PT88: wild type phage ϕ6 containing a host-range mutation on segment M and an engineered mutation (fragment of the *Escherichia coli lacZ* gene for beta-galactosidase) on segment L [49,52]. We mixed the test phage and PT88 at a 1:1 volumetric ratio, and then plated a dilution of this mixture containing ~400 viruses onto a host lawn of PP or ERA. After 24 hr incubation, we harvested and filtered ~400 pfu to obtain a cell free lysate. We tracked the ratio of test virus to PT88 in the starting mixture (*R*_0_) and in the harvested lysate (*R*_1_) by plating on lawns of LM1034: PP containing the complementing fragment of the *E. coli lacZ* gene [52]. LM1034 allows the marked competitor to produce blue plaques on agar containing X-gal (0.4% w/v), whereas unmarked phage produce colorless plaques. We defined fitness (*W*) as the relative change in ratios, *W = R*_1_*/ R*_0_*.* Thus, we assayed fitness on either PP or ERA, but tracked competitor ratios on LM1034 lawns.

## Competing interests

The authors declare that they have no competing interests.

## Authors’ contributions

PT conceived of the study, designed the experiment, performed statistical analysis, and drafted the manuscript. RCM conducted and analyzed the phenotypic assays and follow-up experimental evolution, and helped draft the manuscript. SD conducted the sequence alignment and analyzed the molecular genetic data. RM conducted the ‘known phylogeny’ experimental evolution, and obtained some of the phenotypic data. LSW tested the accuracy of algorithms used in phylogenetic reconstruction, and contributed to the writing. YY and SJL sequenced virus clones. JK provided part of the genomic sequence, analyzed the molecular genetic data, and drafted the manuscript. All authors read and approved the final manuscript.

## Supplementary Material

Additional file 1 Detailed descriptions of the fitness of individual virus clones on Pseudomonas phaseolicola and P. pseudoalcaligenes ERA bacteria, and a supplementary figure showing the experimental design to confirm selection for allele reversion when phage are passaged on P. phaseolicola. Click here for file
