# Supplementary material for: Evolutionary genomics of host-use in bifurcating demes of RNA virus phi-6
Source: BMC Evol Biol. 2012 Aug 22;12:153. doi: 10.1186/1471-2148-12-153 (PMC3495861; doi:10.1186/1471-2148-12-153)
Supplement: Additional file 1 — Detailed descriptions of the fitness of individual virus clones on Pseudomonas phaseolicola and P. pseudoalcaligenes ERA bacteria, and a supplementary figure showing the experimental design to confirm selection for allele reversion when phage are passaged on P. phaseolicola. [file 1471-2148-12-153-S1.docx]

**Evolutionary genomics of host-use in bifurcating demes of RNA virus phi-6**

Paul E. Turner, Robert C. McBride, Siobain Duffy, Rebecca Montville, Li-San Wang, Yul W. Yang, Sun Jin Lee, and Junhyong Kim

*Additional Material*

**Performance of individual clones on PP:** The fitness losses on PP for clones E5 and E7 were likely due to effects of drift, even though their predecessors were equivalent in fitness to the ancestor. Clones F1 thru F8 had immediate predecessors of low fitness on PP, and the continued poor performance of the majority of these strains (i.e., clones F1, F2, F3, F5, F6) may be due to inefficient selection to restore fitness under variable (increasing or decreasing) population size on PP. Fitness restoration on PP shown by clone F7 was likely due to constant selection at large size on this host, and that shown by F8 may be due to correlated improvement on PP despite constant strong selection on ERA. Clone F8 shows positive relative fitness on PP, even though all of its ancestors, E4, D2, and C1, show negative relative fitness. On the other hand, F8 shows considerable increase in relative fitness on ERA compared to its ancestors. One possibility is that the initial growth on ERA involved negative fitness tradeoffs with growth on PP, but with sufficient generations additional fitness gain on ERA involved positive epistatic effects with growth on PP. Last, for clones F9 thru F16, the only viruses which showed low fitness on PP were pairs whose immediate predecessors were also of low fitness on this host (i.e., F9-F10, F13-F14).

**Performance of individual clones on ERA:** Of the clones that were descendents of clones D1 and D2, the majority of these maintained the ability to infect ERA shown by their immediate predecessors (but see clones F1, F6; Table 1); all such clones that could infect ERA were more fit than B1 on this host, including clones F1-F4 which experienced variable selection imposed on PP.

Clone F7 showed strong performance on ERA, even though it recently experienced selection at large size on PP; these combined data suggested that selection on PP led to further (correlated) improvement on ERA for this lineage. We infer that the observed P3 mutations in clones F1 and F6 caused them to lose ability to infect ERA. Of clones F9 thru F16, only three viruses showed ability to infect ERA but these clones were more fit on ERA than clone B1. Similar to the above mentioned outcomes, clone F12 maintained the strong growth on ERA seen in its predecessor, despite recent variable selection on PP. High fitness of clone F15 on ERA was the only puzzling outcome, given its immediate predecessor showed inability to infect this host. It is likely that clone F15 happens to harbor a spontaneous mutation for ERA infection even though it was not recently subjected to selection on this host. Examining the sequence of mutations, the C2 clone in the PP clade acquired an amino-acid change (V131S) in the S segment P12 envelope protein, which potentially caused loss of growth on ERA hosts. The F15 lineage acquired four spontaneous amino-acid changes: T137I in L-P1, E8D in M-P3, N118S in S-P12, and W211C in S-P5. One possibility is that the mutation in S-P12, an Asn to Ser change, synthetically suppressed the non-growth phenotype of the V131S S-P12 mutation in the C2 clone. Two other protein mutations are notable in this line. The W211C mutation in S-P5 is a parallel mutation to the same mutation found in line C1. Clone C1 had a +0.936 change in relative fitness compared to the B1 clone, which is one of the largest gains in relative fitness for any of the lineages. In addition, a mutation in M-P3 protein is found in clone E4 (G265A) and clone F8 (E374Q), two lines with 3^rd^ and 4^th^ largest fitness gains in the ERA host condition. Thus, the E8D mutation in M-P3 may also confer large fitness gains on the ERA host. In sum, the unexpectedly large fitness gain for F15 clone may be due to large fitness effect variants on the ERA host that arose through chance *de novo* mutations or were present as low frequency segregating variants in PP.

**Performance of individual clones relative to their immediate predecessors:** All six clones isolated after treatments with increasing population sizes on PP showed higher mean ln*W* values than their predecessors (cf., Fig. 1, Table 1), but only three of these clones (F1, F9, F13) statistically improved in PP growth. These results suggested that exposure to increasing population sizes on PP allowed selection to foster adaptation on this host. Most of the viruses in this treatment failed to infect ERA or showed equivalent growth as their predecessor; only clone F5 significantly improved in growth on ERA (Table 1), suggesting a correlated response to selection because the clone showed higher (but non-significant) improvement on PP.

For the six clones drawn from treatments with decreasing population sizes on PP, mean ln*W* values on PP were measurably equal or higher than their predecessors in five cases, and four outcomes were statistically significant (Table 1). These data suggested that the presumed decreasing efficiency of selection still allowed populations to improve in PP growth. Only clone F2 in this treatment group improved in growth on ERA, perhaps due to correlated selection in association with increased PP growth. One possibility for asymmetry in the phenotypic evolution between the increasing versus decreasing population size treatments is that selection operated on segregating variance from the initial populations rather than *de novo* mutations in later populations. Thus, having a larger initial population size might have resulted in larger initial variance for selection.

None of the six bottlenecking treatments allowed mean ln*W* to significantly improve PP, and in two cases (E5, E7) this treatment produced a significant fitness decline relative to the predecessor (Table 1). Also, these bottlenecked lineages failed to improve on ERA, or could not infect the host at all.

**Additional Figure 1 Design for additional experimental evolution examining effects of population size on fixation of a mutation inferred to contribute to host-specific fitness of phage φ6.**
